# Chromosome Y variants from different inbred mouse strains are linked to differences in the morphologic and molecular responses of cardiac cells to postpubertal testosterone

**DOI:** 10.1186/1471-2164-10-150

**Published:** 2009-04-07

**Authors:** Bastien Llamas, Ricardo A Verdugo, Gary A Churchill, Christian F Deschepper

**Affiliations:** 1 Experimental Cardiovascular Biology Research Unit, Institut de recherches cliniques de Montréal (IRCM) and Université de Montréal, 110 Pine Ave West, Montréal (QC), H2W 1R7, Canada; 2 The Jackson Laboratory, 600 Main Street, Bar Harbor, ME 04609, USA; 3 Present address: Australian Centre for Ancient DNA, University of Adelaide, SA 5005, Australia

## Abstract

**Background:**

We have reported previously that when chromosome Y (chrY) from the mouse strain C57BL/6J (ChrY^C57^) was substituted for that of A/J mice (ChrY^A^), cardiomyocytes from the resulting "chromosome substitution" C57BL/6J-chrY^A ^strain were smaller than that of their C57BL/6J counterparts. In reverse, when chrY^A ^from A/J mice was substituted for that of chrY^C57^, cardiomyocytes from the resulting A/J-chrY^C57 ^strain were larger than in their A/J counterparts. We further used these strains to test whether: 1) the origin of chrY could also be linked to differences in the profile of gene expression in the hearts of adult male mice, and 2) post-pubertal testosterone could play a role in the differential morphologic and/or molecular effects of chrY^C57 ^and chrY^A^.

**Results:**

The increased size of cardiomyocytes from adult male C57BL/6J mice compared to C57BL/6J-chrY^A ^resulted from the absence of hypertrophic effects of post-pubertal testosterone on cells from the latter strain. However, gene profiling revealed that the latter effect could not be explained on the basis of an insensitivity of cells from C57BL/6J-chrY^A ^to androgens, since even more cardiac genes were affected by post-pubertal testosterone in C57BL/6J-chrY^A ^hearts than in C57BL/6J. By testing for interaction between the effects of surgery and strain, we identified 249 "interaction genes" whose expression was affected by post-pubertal testosterone differentially according to the genetic origin of chrY. These interaction genes were found to be enriched within a limited number of signaling pathways, including: 1) *p53 signaling*, which comprises the interacting genes *Ccnd1*, *Pten *and *Cdkn1a *that are also potential co-regulators of the androgen receptors, and 2) *circadian rhythm*, which comprises *Arntl/Bmal1*, which may in turn regulate cell growth via the control of *Cdkn1a*.

**Conclusion:**

Although post-pubertal testosterone increased the size of cardiomyocytes from male C56BL/6J mice but not that from their C57BL/6J-chrY^A ^counterparts, it affected gene expression in the hearts from both strains. However, several cardiac genes responded to post-pubertal testosterone in a strict strain-selective manner, which provides possible mechanisms explaining how chrY may, in part via interference with androgen regulatory events, be linked to morphologic differences of cardiac cells of adult male mice.

## Background

Chromosome Y (chrY) stands out from all other chromosomes: it is comprised for most of its length of sequences that do not recombine (the so-called "male-specific part"), it contains many repetitive DNA sequences of unclear significance, and it harbors only a small number of active genes [1]. Genes from the male-specific part of chrY are clearly not essential for life, as demonstrated in female organisms. Besides *Sry *(the master gene in sex determination), most of what is known about the functions of other chrY genes relates to their effects on male reproductive and sex accessory organs [2-4]. Nonetheless, genetic variants of chrY have been shown to have an impact on functions unrelated to male reproductive biology, including the severity of experimental allergic encephalomyelitis in mice [5], the incidence of prostate cancer in humans [6,7], and several cardiovascular conditions such as hypertension and high plasma cholesterol in either humans or animal models [8-10]. The mechanism explaining these particular effects of chrY genes are still unclear, and cannot be explored by using classical genetic methods because chrY genes do not recombine.

Recently, we have reported that chrY accounts for a large part of the genetic variance of the size of adult male heart muscle cells [i.e. cardiomyocytes (CMs)] isolated from mice derived from crosses between A/J and C57BL/6J [11]. In agreement with the linkage mapping results, we also found that when chrY from the parental strain C57BL/6J (ChrY^C57^) was substituted for that of A/J mice (ChrY^A^), CMs from the resulting "chromosome substitution" (or "consomic") C57BL/6J-chrY^A ^strain (C57.Y^A^) were smaller than that of their C57BL/6J counterparts. In reverse, when chrY^A ^from A/J mice was substituted for that of chrY^C57^, CMs from the resulting A/J-chrY^C57 ^strain (A.Y^C57^) were larger than in their A/J counterparts. Since the only genetic differences in these unique animal models are those possibly existing between C57BL/6J and C57.Y^A^, we used these strains to further test whether, in addition to affecting the size of CMs, the origin of chrY could also be linked to differences in the profile of gene expression in the hearts of adult male mice. Moreover, since: 1) one of the most obvious consequences of chrY is to foster the development of male gonads and the production of testosterone, and 2) there is preliminary evidence that chrY polymorphisms may interfere with some biologic effects of testosterone [10,12], we tested whether postpubertal testosterone could play a role in the differential morphologic and/or molecular effects of chrY^C57 ^and chrY^A ^on cardiac cells.

## Results and discussion

**Figure 1:**
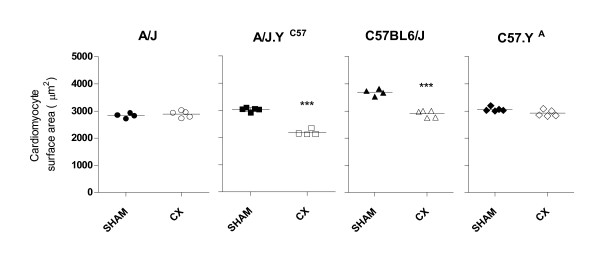
**Surface area of cardiomyocytes from 12 week-old male A/J, C57BL/6J, A/J.Y^C57 ^or C57.Y^A ^mice**. All animals have undergone surgery (either sham or castration) at 4 weeks of age. The figures represent values for individual animals scattered around the mean value of the group. ***P < 0.001 vs. sham-operated.

**Figure 2:**
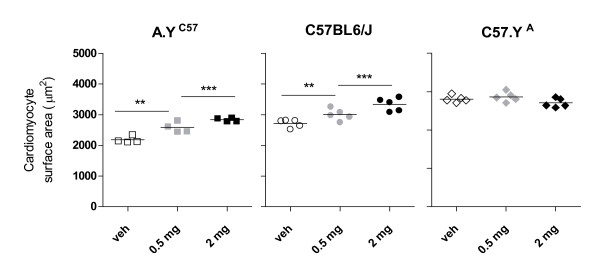
**Surface area of cardiomyocytes from 12 week-old male A/J.Y^C57^, C57BL/6J or C57.Y^A ^mice**. All animals have undergone surgery (either sham or castration) at 4 weeks of age, and were treated with testosterone cypionate (either 0.5 mg or 2 mg/kg body weight) from 8 to 12 weeks of age. The figures represent values for individual animals scattered around the mean value of the group. **P < 0.01; ***P < 0.001.

In extension to our previous findings showing that CMs from strains carrying chrY^C57 ^were larger than that from their counterparts carrying chrY^A ^[11], we found that prepubertal castration (which prevents the increase in plasma testosterone that normally occurs at puberty) decreased the size of CMs in adult animals from strains carrying Y^C57 ^(i.e. C57BL/6J and A.Y^C57 ^mice), but not in their counterparts carrying chrY from A/J (Y^A^), i.e. A/J and C57.Y^A ^mice (Figure 1). The effect of castration (CX) was not the result of testosterone production being higher in C57BL/6J mice, as plasma testosterone in adult male C57BL/6J mice (2.5 ± 1.7 ng/ml) was in fact lower than in their A/J (10.8 ± 3.6) or C57.Y^A ^(9.6 ± 4.5) counterparts, this result being in agreement with another report showing that the levels of plasma testosterone are lower in male C57BL/6J mice than in several other mouse inbred strains [13]. Likewise, the abundance of androgen receptor mRNA and protein was similar in the hearts of all strains (not shown). However, chronic treatment of mice that had been castrated at 3 weeks of age with 2 dosages of testosterone from 8 to 12 weeks of age increased the size of CMs of C57BL/6J in a dose-dependent fashion, but not in the consomic strain C57.Y^A ^(Figure 2). In contrast, testosterone also increased the size of CMs from consomic A.Y^C57 ^in a dose-dependent fashion, although we had found that cardiomyocytes from the parental A/J strain were not affected by CX. Altogether, these results showed that the difference in size of CMs from intact (sham-operated) C57BL/6J and C57.Y^A ^male mice resulted from the fact that cardiomyocytes from C57.Y^A ^were not sensitive to the hypertrophic effects of post-pubertal testosterone.

**Figure 3:**
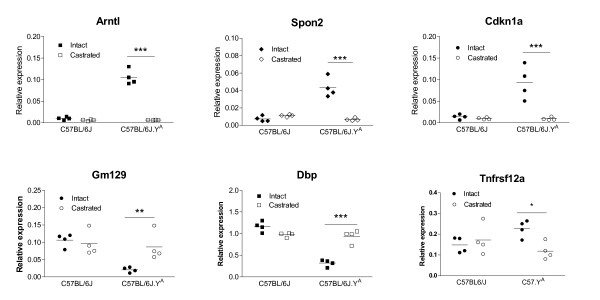
**Real-time RT-PCR quantifications of 6 genes in the hearts of adult male C57BL/6J and C57.YA (either intact sham-operated of castrated)**. *P < 0.05; **P < 0.01; *P < 0.001 (Bonferroni-corrected post-hoc tests).

We next compared the cardiac transcriptosomes of 4 sham-operated and 4 CX adult male mice from the C57BL/6J and C57.Y^A ^strains. The numbers of probes and corresponding genes showing differential expression between groups (selected on the basis of a FDR < 0.1) are summarized in Table 1. We found that the effects of post-pubertal testosterone and/or the origin of chrY affected the profile of cardiac gene expression in a manner that was very different than their respective effects on the size of CMs. For instance, although post-pubertal testosterone had no effect on the size of CMs from C57.Y^A ^male mice, it affected the expression level of many cardiac genes in that strain, and even of a greater number of genes than in their C57BL/6J counterparts. This indicated that the lack of hypertrophic effects of post-pubertal testosterone on CMs from C57.Y^A ^was not the manifestation of an insensitivity of cells from this strain to androgens, but rather the result of a differential molecular response to the hormone. Likewise, despite the fact that the effect of chrY on the size of CMs was observed only in sham intact C57BL/6J mice but not in their CX counterparts, more cardiac genes showed chrY-dependent differential expression in CX than in sham intact animals. This indicated that the presence of post-pubertal testosterone was not required in order to observe an effect of chrY. Finally, in addition to the marginal effects of strain and surgery, we also tested for interaction between these two factors, which allowed us to identify within the genome 249 unique genes (hereafter referred to as "interaction genes") whose expression was affected by CX (and thus by post-pubertal testosterone) differentially according to the genetic origin of chrY (Additional file 1). Most interactions genes showed rather large differences in gene expression, as ~44% of them displayed either strain-dependent differences or surgery-dependent changes were greater than 1.5 fold. To validate the results of the microarray analysis, we performed real-time RT-PCR quantifications for 6 of the interaction genes. The differences detected by RT-PCR were similar to those that had been detected with the microarray (Figure 3).

**Table 1:** Numbers of probes and corresponding unique genes in different conditions

**Condition**	**Probes**	**Corresponding unique genes**
Present on Mouse Ref-8	25,697	18,118
Detected (in at least one out of four conditions)	12,764	8,297
Surgery: overall effect	3,030	2,274
Surgery: effect in C57BL/6J	1,103	845
Surgery: effect in C57.Y^A^	2,706	2,042
Strain: overall effect	3,282	2,508
Strain: effect in CX	3,209	2,421
Strain: effect in sham (intact)	479	381
Interaction between surgery and strain	318	249

**Figure 4:**
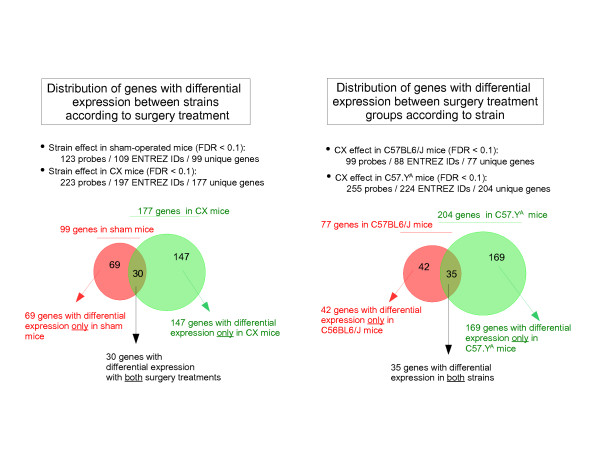
**Venn diagrams showing the distribution of "interaction genes" within the 2 particular surgery groups or within the 2 particular strains used in the current study (n = 4 per group)**.

To further determine 1) which particularly surgery group comprised genes where the strain-dependent differences in expression varied according to the surgery status, and 2) to which particular strain corresponded genes where the effect of CX varied according to the origin of chrY, we performed post-hoc analyses on the hybridization results obtained with probes from all interaction genes. Accordingly, we found that the expression of interaction genes was differentially affected by strain to a greater extent in CX (177/249 genes) than in intact sham (99/249 genes) mice (with 3 genes where the effect of surgery treatment did not reach significance with the post-hoc analysis) (Figure 4). Likewise, genes responded to CX in a strain-specific manner to a greater extent in C57.Y^A ^(204/249 genes) than in C57BL6/J (77 genes/249) mice (with also 3 genes where the effect of strain did not reach significance with the post-hoc analysis) (Figure 4).

In order to assess the biological significance of the interaction genes, we performed a Set Enrichment Test for every pathway in the Kyoto Encyclopedia of Genes and Genomes (KEGG) [14] to calculate the probability of observing a given number of selected genes in a pathway by chance. The six following KEGG pathways showed enrichment for interaction genes using a p-value ≤ 0.05 as a criteria for selection: *circadian rhythm *[containing *Arntl1 (also known as Bmal1), Per2, Per3 *and *Cry2*], *p53 signaling pathway *(containing *Cdkn1a, Gadd45g, Ccnd1 *and *Pten*), *melanoma *(also containing *Cdkn1a, Ccnd1 *and *Pten*, as well as *Fgf7*), *cytokine-cytokine receptor interaction *(containing *Acvr2b, Ccl8, Tnfrsf12a, Cxcl1 *and *IL28ra*), *leukocyte transendothelial migration *(containing *Myl7, Cldn5, Jam5 and Vcam1*), and *tight junction *(also containing *Myl7 *and *Cldn*5, as well as *Jam2, Pten and Myh7*) (see Additional file 2 and Table 2). However, it should be noted that there is some overlap between the groups of genes assigned by KEGG to each pathways: *melanoma *shares 5 genes with either *p53 signaling pathway *or *cytokine-cytokine receptor interaction*, it also shares 3 genes with *tight junction*, and the latter shares 20 genes with *leukocyte transendothelial migration*. This is likely to result from the fact that the KEGG classification is somewhat arbitrary, and sometimes assigns genes on the basis of particular biologic manifestations rather than regulatory pathways or functions. Nonetheless, overlap between KEGG pathways suggests that a common biological process underlies the selection of these particular genes. Of note, one additional limitation of the Set Enrichment Test is that the coverage of KEGG pathways is still incomplete at the current time. For instance, although *Dbp, Tef, Nr1d2 *and *Rora *are all well-known downstream effectors of clock genes [15,16], none of them have been included yet within the KEGG *circadian rhythm *pathway. Likewise, *Myl4 *has not been assigned to either *leukocyte transendothelial migration *or *tight junction *despite being highly homologous to *Myl7*. Interestingly, 14 out of the 17 interaction genes enriched within KEGG pathways display strain-dependent differences or surgery-dependent changes that were greater than 1.5 fold (see Additional file 2), which may constitute one other indication that these particular genes play biologically important roles.

**Table 2:** Selected KEGG pathways showing enrichment for interaction genes

**KEGG ID**	**Term**	**Size**	**Expected Count**	**Count**	**P-value**
4710	Circadian rhythm	10	0.33	6	1.8E-07
4115	p53 signaling pathway	31	1.02	5	0.0028
5218	Melanoma	32	1.05	4	0.0190
4060	Cytokine-cytokine receptor interaction	70	2.30	6	0.0249
4670	Leukocyte transendothelial migration	52	1.71	5	0.0257
4530	Tight junction	73	2.40	6	0.0299

Listed KEGG pathways correspond to those showing significant enrichment (p < 0.05) for "interaction genes". *Size *corresponds to the number of genes assigned by KEGG within each particular pathway. *Expected count *corresponds to the number of interaction genes could be expected to be found if there was no significant enrichment, *count *corresponds to the effective number of genes showing significant enrichment (p < 0.05) (see also Additional file 2).

**Figure 5:**
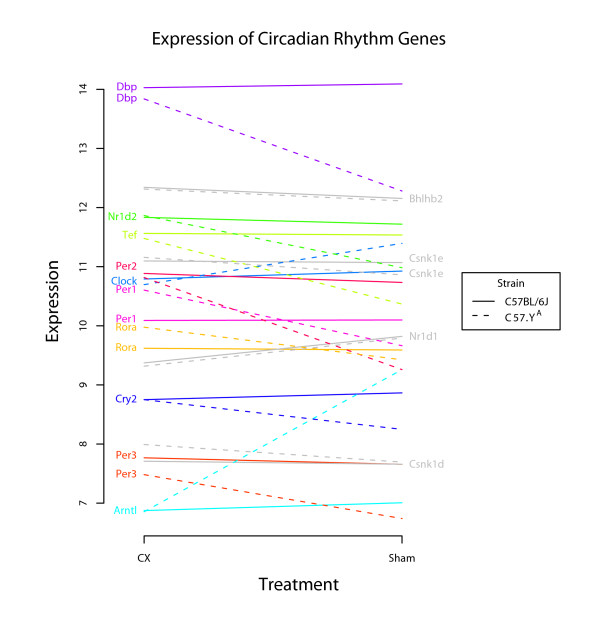
**Probe expression pattern for circadian genes (either genes listed in the KEGG pathway mmu04710 *circadian rhythm *or interaction genes described as circadian in the literature)**. Each line connects average expression of a given gene between CX and sham operated mice. Strain of mice is denoted by a solid line, for C57BL/6J and a dashed line for C57.Y^A^. The vertical scale corresponds to log_2 _(hybridization intensity value). For the 8 genes listed in the left part of the figure (with a different color for each symbol and corresponding lines), the difference between the slopes of the lines reflects the interaction effects. For 4 additional genes (listed in the right part of the figure, with symbols and corresponding lines formatted in gray), there is no significant interaction effect.

**Figure 6:**
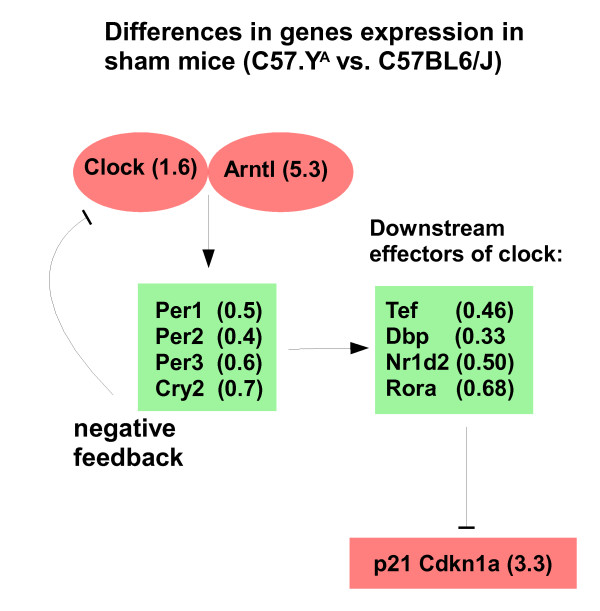
**Diagram summarizing circadian clock genes that are differentially expressed in hearts from intact sham-operated C57BL/6J and C57.Y^A ^mice**. Boxes are in red or green when gene expression is higher in intact male C57.Y^A ^or C57BL/6J mice, respectively. Lines terminating with an arrowhead indicate positive regulation; those terminating with a bar indicate negative regulation. The values represent the ratio of mRNA abundance in intact (sham-operated) male C57.Y^A ^vs. C57BL/6J hearts.

*Circadian rhythm*, in addition to being *the *KEGG pathway showing the most significant enrichment for interactions genes, also comprised genes showing some of the largest differences in expression among interaction genes. If we consider all 14 genes that could be classified as "circadian" on the basis of both KEGG pathway database and additional annotation from the literature, we found that expression of 14 of them were significantly affected by CX in C57.Y^A ^mice in contrast to only one gene in C57BL/6J (Table 3 and Figure 5). Although only 10 genes were included in the interaction genes set, the post-hoc tests revealed evidence for interaction in two extra circadian genes, i.e. Csnk1e and Csnk1d, that showed 20% increase in castrated animals but only in the C57.Y^A^strain. As a consequence of this differential response to post-pubertal testosterone, most circadian genes showed strain-dependent differences in cardiac expression between intact C57BL/6J and C57.Y^A ^mice, but not in their CX counterparts (Figure 6). One caveat concerning these experimental results is that expression of circadian genes is, by definition, very sensitive to time. Although we took care of killing all mice at the same time of day (between 9:00 and 10:00 AM), further validation requires additional experiments using mice from both strains killed at different times of the 24-hour nyctemer. Nonetheless, there are currently other lines of evidence suggesting that the current results are biologically pertinent, since: 1) recent reports showed that androgens are major regulators of clock genes in C57BL/6J mice and that they are responsible for sex-dependent differences in circadian activity [17,18], and 2) male C57BL/6J mice do not show (in contrast to male A/J counterparts) circadian oscillations of heart rate (corrected for locomotor activity) [19]. Moreover, it has also been reported recently that *Arntl/Bmal1 *is a major regulator of *Cdkn1a *(whose expression is also greatly affected by CX in a C57.Y^A^-specific fashion, as discussed below) and that *Arntl*-dependent changes in the concentration of the corresponding p21Waf1/Kip1 protein correlate with changes in the rate of liver cell proliferation, in keeping with the known functions of this cyclin inhibitor [16].

**Table 3:** Summary of results concerning circadian genes

		**C57BL/6J**		**C57.YA**		**Interaction**
				
**Gene Symbol**	**Gene Name**	**FC**	**FDR**	**FC**	**FDR**	**FDR**
Arntl	aryl hydrocarbon receptor nuclear translocator-like	-1.10	0.531	-5.30	**0.000**	**0.006**
Dbp	D site albumin promoter binding protein	-1.05	0.772	2.94	**0.000**	**0.006**
Tef	thyrotroph embryonic factor	1.02	0.851	2.16	**0.001**	**0.008**
Per2	period homolog 2 (Drosophila)	1.11	0.531	2.95	**0.001**	**0.009**
Cry2	cryptochrome 2 (photolyase-like)	-1.08	0.370	1.42	**0.001**	**0.019**
Clock	circadian locomoter output cycles kaput	-1.10	0.284	-1.62	**0.001**	**0.024**
Nr1d2	nuclear receptor subfamily 1, group D, member 2	1.08	0.561	1.84	**0.001**	**0.033**
Per3	period homolog 3 (Drosophila)	1.08	0.503	1.67	**0.001**	**0.034**
Rora	RAR-related orphan receptor alpha	1.02	0.845	1.46	**0.002**	**0.081**
Per1	period homolog 1 (Drosophila)	-1.01	0.923	1.92	**0.004**	**0.087**
Csnk1e	casein kinase 1, epsilon	1.02	0.826	1.23	**0.016**	0.230
Csnk1d	casein kinase 1, delta	1.04	0.789	1.23	**0.061**	0.437
Nr1d1	nuclear receptor subfamily 1, group D, member 1	-1.36	**0.017**	-1.39	**0.006**	0.920
Bhlhb2	basic helix-loop-helix domain containing, class B2	1.14	0.251	1.15	0.135	0.942

Genes were considered as "circadian" on the basis of information from both the KEGG pathway database and additional annotation from the literature. FC: fold change of CX over Sham operated animals; FDR: P-values for the interaction between strain and treatment effects after adjustment for multiple comparisons with an FDR transformation (see methods); FDR values < 0.1 are formatted in bold.

**Figure 7:**
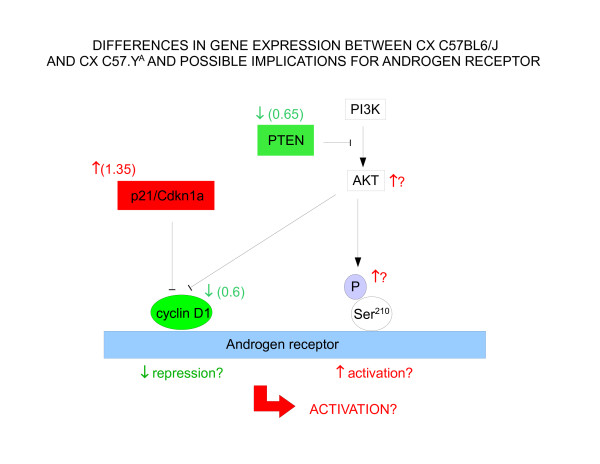
**Diagram summarizing differences in expression of interaction genes enriched within the KEGG p53 signaling pathway, and possible interactions of corresponding proteins with the androgen receptor**. Boxes are in red or green when gene expression is increased or decreased in CX male C57BL/6J mice, respectively. Lines terminating with an arrowhead indicate positive regulation, those terminating with a bar indicate negative regulation. The values represent the ratio of mRNA abundance in CX male C57BL/6J vs C57.Y^A ^hearts.

Within the *p53 signaling pathway *(second in importance in terms of enrichment for interacting genes), *Ccnd1*, *Pten *and *Cdkn1a *may be of particular interest. Indeed, *Ccnd1 *encodes for cyclin D1 which is considered as one of the *few bona fide *AR-specific co-repressors, both in androgen-dependent and-independent environments [20-22]. Cyclin D1 (down-regulated in CX C57BL/6J vs. CX C57.Y^A^) can be silenced by Akt [23], and the latter may be more active in CX C57BL/6J because its inhibitor *Pten *is down-regulated vs. CX C57.Y^A^. Cyclin D1 can also be silenced by the p21Waf1/Kip1 cyclin inhibitor protein (encoded by *Cdkn1a*) [24], the latter being upregulated in CX C57BL/6J vs. CX C57.Y^A^. Finally, an active Akt pathway may stimulate the androgen pathway in part via phosphorylation of the androgen receptor [25]. If all these strain-dependent differences in gene expression in CX animals translate into changes at the protein level, the balance between co-repressors and co-activators would tip towards the latter in CX male C57BL/6J mice (Figure 7). Of note, some degree of constitutive activation of the AR in CX male C57BL/6J might explain why fewer genes respond to post-pubertal T in this strain than in C57.Y^A ^mice, and why there are fewer differences in gene expression in intact C57BL6/J and C57.Y^A ^than in their CX counterparts. Likewise, increased sensitivity of the AR might explain why plasma testosterone is lower in intact C67BL6/J than in C57.Y^A ^mice, as measured by ourselves (data not shown) and in agreement with a previous report [13].

Expression of *Cdkn1a *is increased ~5-fold in the hearts of intact vs. CX C57.Y^A ^(see Additional file 1), which is in keeping with previous reports showing that this gene is an important target of activated androgen receptors [26]. However, there was also striking strain-selectivity in the response, as there are no changes in expression between intact and CX C57BL/6J mice. Interestingly, *Cdkn1a *(which encodes for a well-known regulator of cell growth) has been shown to escape from androgen-dependence in some types of prostate cancers, and this phenomenon has been implicated as one of the possible factors leading to an increase in the growth properties of these cells [27-29]. Although cardiac cells are post-mitotic, p21 may act on these cells as a negative regulator of hypertrophy [24,30]. The lack of response of *Cdkn1a *to post-pubertal testosterone in C57BL/6J might thus provide an explanation for why pubertal androgens increase the size of cardiomyocytes in this strain, but not in C57.Y^A^.

**Figure 8:**
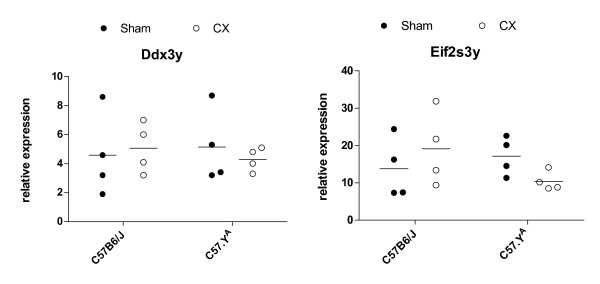
**Real-time RT-PCR quantifications of the abundance of mRNA transcripts of Ddx3 and Eif2s3y in the hearts of adult male C57BL/6J and C57.YA (either intact sham-operated of castrated)**. The figures represent values obtained with samples from individual animals, scattered around the mean of the group.

**Figure 9:**
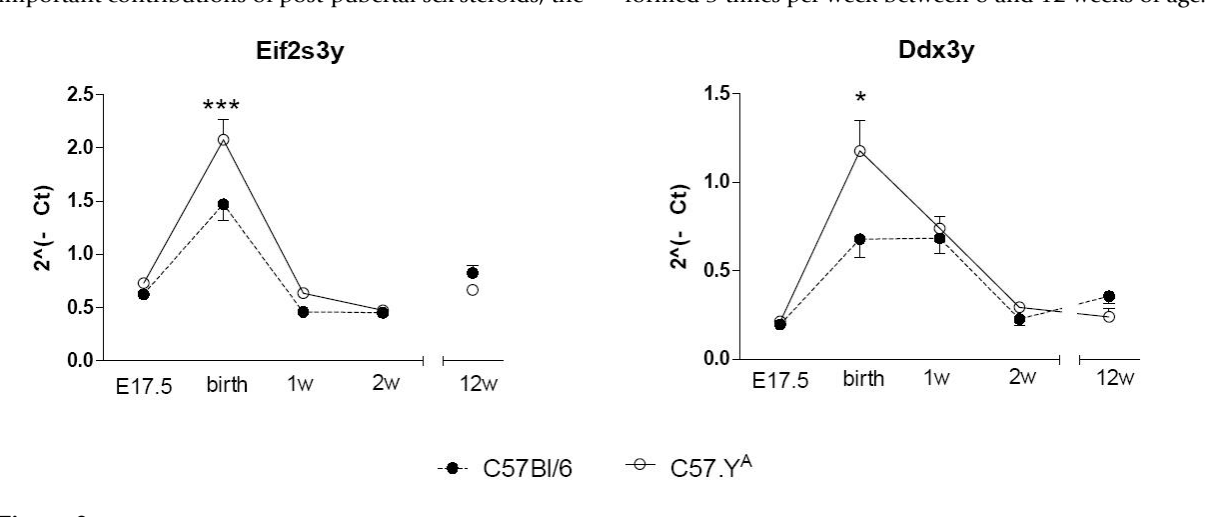
**Real-time RT-PCR quantifications of the abundance of mRNA transcripts of Ddx3 and Eif2s3y in the hearts of intact male C57BL/6J and C57.Y^A ^taken at different times during development**. The points represent means ± SD (n = 5).

Thirteen genes have been reported on the male-specific portion of mouse chrY. The short arm of mouse chrY (Yp, ~2.5 Mb) contains the best characterized sequences to date, i.e. *Sry *and the 9 following genes: *Ube1y1, Zfy1, Zfy2, Jarid1d, Eif2s3y, Uty, Ddx3y, Usp9y and Rbmy1a1 *[31]. Three other genes, i.e. *Ssty1, Ssty2 and Sly*, are present on the long arm (Yq) in multiple copies [32,33]. Out of these 13 genes, we found only 5 of them to be expressed in hearts, i.e. *Ddx3y, Uty, Eif2s3y, Jarid1d *and *Sly *(data not shown). These genes appear to be expressed in a ubiquitous fashion, as we have detected by RT-PCR corresponding amplification products in many other non-cardiac tissues (listed below in the Methods section; data not shown). The first four of these genes were present on the Illumina MouseRef-8 v2.0 BeadChip, whose analysis revealed possibly significant differences among experimental groups. For *Ddx3y*, the effect of strain was significant (P < 0.05), but not that of surgery nor the interaction between the two. For *Eif2s3y*, the interaction (but not strain or surgery) was significant (P < 0.01). However, quantification of the level of expression of *Ddx3y *and *Eif2s3y *in the same samples by quantitative RT-PCR did not reveal any significant effect of strain or surgery in hearts from adult mice (Figure 8). We also measured by quantitative RT-PCR the level of expression of all 5 genes in hearts of C57BL.6J and C57.Y^A ^mice at different times during development. At the time of birth (but at no other tested time), both *Ddx3y *and *Eif2s3y *showed lower levels of expression in hearts from C57BL/6J male mice compared to their C57.Y^A ^counterparts (Figure 9). However, no polymorphism was found between the 2 strains for the regions including ~1 kb of upstream promoter and each exon (including 30 bp of intron flanking sequences as well as ~1 kb of the 3'-untranslated region). It therefore remains to be determined whether the perinatal differences in expression of these two chrY genes may relate to differences in the effects of post-pubertal testosterone during adult life, and if so, by which mechanism. Likewise, which polymorphisms on chrY may be responsible for such differences needs additional sequencing.

## Conclusion

The increased size of cardiomyocytes from adult male C57BL/6J mice compared to C57BL/6J-chrY^A ^resulted from the absence of hypertrophic effects of post-pubertal testosterone on cells from the latter strain. However, gene profiling revealed that the lack or hypertrophic effects could not be explained on the basis of an insensitivity of cells from C57BL/6J-chrY^A ^to androgens, because post-pubertal testosterone affected the expression of more genes in hearts from in C57BL/6J-chrY^A ^than in that from C57BL/6J. Moreover, several cardiac genes responded to post-pubertal testosterone in a strict strain-selective manner. These findings provide possible mechanisms explaining how chrY may, in part via interference with androgen regulatory events, be linked to morphologic differences of cardiac cells of adult male mice. Of note, there are well-known differences in the manifestations of cardiovascular diseases between men and women [34,35]. Despite the important contributions of post-pubertal sex steroids, the latter do not account for all differences, and there is now evidence that genes present on sex chromosomes also play important roles as well [36]. In light of the current data, it becomes worth testing further to which extent: 1) chrY related associations can be explained in terms of modulation of the effects of androgens, and 2) chrY may participate to male/female differences in cardiovascular diseases.

## Methods

### Experimental animals

All procedures on animals were approved by the Institut de Recherches Cliniques de Montréal (IRCM) Institutional Animal Care Committee and conducted according to guidelines issued by the Canadian Council on Animal Care. All mice were obtained from The Jackson Laboratory (Bar Harbor, ME). In addition to the parental A/J and C57BL/6J strains, we used their respective chromosome-substitution strains (also known as consomic strains) A/J-chrY^C57BL/6J^/NaJ and C57BL/6J-chrY^A/J^/NaJ strains (previously simply identified as A.Y^C57 ^and C57.Y^A^). The latter corresponded to either A/J or C57BL/6J male mice whose chrY has been switched by inbreeding for that of the other strain [37]. To evaluate the effect of post-pubertal testosterone, all animals were operated between 3 and 4 weeks of age, with castration being performed in some mice to prevent the rise of testosterone that normally occurs at puberty, and sham-operated mice being used as controls. To evaluate the effects of testosterone replacement, all animals were castrated between 3 and 4 weeks of age, and subcutaneous injections of either vehicle (i.e. vegetable oil) or testosterone cypionate (0.5 and 2 mg/kg) were performed 3 times per week between 8 and 12 weeks of age.

### Isolation of CMs and videomicroscopy

CMs were isolated from 12 week-old mouse hearts mounted for cardiac retrograde aortic perfusion as performed previously [11,38,39]. After fixing, the profiles of isolated CMs were visualized by autofluorescence (excitation 540 nm, emission 620 nm) and their image captured with a Retiga EXi monochrome camera (Qimaging, Surrey, BC, Canada). The area, length, width and width/length ratio of ~100 CMs per heart were measured using the public domain Java image processing program ImageJ [40], as described previously [11].

### Microarray experiments

Total RNA was extracted, using the *RNeasy minikit *(Qiagen Canada, Mississauga, ON), from cardiac left ventricles obtained from 12 week-old male C57BL/6J or C57.Y^A ^that had undergone either castration or sham surgery between 3 and 4 weeks of age. All animals were killed between 9:00 and 10:00 AM. Four animals were used for each group, thus providing a total of 16 individual samples. Biotinylated probes were prepared from 50 ng of total RNA, using the Ambion Illumina TotalPrep RNA Amplification kit (Applied Biosystems, Streetsville, ON). The probes were hybridized to two Illumina MouseRef-8 v2.0 BeadChips that carry 8 microarrays each, containing 25,697 probes covering 19,728 non-redundant, well-annotated RefSeq sequences covered by either one single probe (79.3%), 2 probes (15%) or 3 or more probes (5.7%). Raw data (probe unnormalized intensity values) were obtained using the BeadStudio software (Illumina, San Diego, CA) and imported into the R 2.7.0 (UNIX) language/environment for normalization and analysis. Quantile normalization was performed using functions provided by the Affy package, as described [41]. Raw and normalized data have been deposited into the Gene Expression Omnibus (GEO) public depository (submission GSE15354), in accordance with MIAME standards.

To decrease the number of multiple comparisons across genes, undetected genes were eliminated from the dataset using Present/Absent calls, as recommended [42]. Detection probability was estimated with the BeadStudio application by using the distribution of intensity values of negative probes. Transcripts were called as present when probability of detection was equal or higher than 0.96. Probes having a "present" call in at least 50% of samples belonging from any strain and/or surgery group were selected for further analyses. A linear model including genotype (strain) and treatment (castration) was fitted to normalized data: *y*_*ij *_= *μ *+ *G*_*i *_+ *T*_*j *_+ *I(GxL)*_*ij *_+ *e*_*ijk*_, where *y*_*ij *_is the normalized-transformed gene expression, *μ *is the population mean, *G*_*i *_is the effect of i^th ^genotype, *T*_*j *_is the effect of j^th ^castration treatment, *I(GxT)*_*ij *_is the effect of genotype by treatment interaction, and *e*_*ijk *_is the residual effect. The significance of effects from strain, surgery and their interaction was tested by appropriate contrasts in a *F-test *between groups in a factorial ANOVA design. The F-tests were calculated with the James-Stein shrinkage estimate using information from neighboring probes [43]. P-values were calculated by performing 1000 permutation of samples to break their association to expression values, then corrected for multiple comparisons by adaptive false-discovery rate (FDR) transformation [44], using a 10% FDR cutoff. All computations were done with the R/Maanova package v. 1.10.0 [45].

The set of probes with significant interaction effects, i.e. the "Interaction Genes set" was tested for enrichment of any KEGG pathway in the KEGG database . Enrichment for KEGG pathways was tested by comparing the number of genes in a pathway to what would be expected by chance if the Interacting Genes set was a random sample from the list of genes present in samples. Using a hypergeometric function, this probability is calculated, which is equivalent to a Fisher's Exact Test. Genes with multiple probes in the microarrays were selected if at least one probe presented an FDR < 0.1 for the interaction term. Genes with multiple probes were counted only once in this analysis. Pathways with a p-value ≤ 0.05 were selected. Computations were performed with the *GOstats *package for R [46].

### PCR and RT-PCR amplification of total RNA

To verify which chrY genes were expressed in the heart and/or other tissues, total RNA was extracted from several tissues of adult C57BL/6J mice using the *RNeasy minikit *(Qiagen Canada, Mississauga, ON). The tissues were the left cardiac ventricles, quadriceps muscle, small and large intestine, brain, thymus, lungs, kidneys, pancreas, adrenal glands, testes, peritoneal fat and bladder. Complementary DNA was then synthesized (using 2 *μ*g of total RNA from each tissue) using *Superscript II reverse transcriptase *(Invitrogen Canada, Burlington, ON), and 100 ng of cDNA from each sample was then PCR amplified. Each primer pairs was designed to overlap at least one exon (with the exception of Sry, which is comprised of a single exon). Amplification products were visualized by UV illumination of products electrophoresed in an agarose gel containing ethidium bromide. Primers used for amplification of chrY of cDNA reverse-transcribed from chrY gene mRNA transcripts are summarized in Additional file 3.

Quantitative RT-PCR was performed to: 1) compare the abundance of chrY transcripts in hearts from C57BL/6J and C57.YA male mice; and 2) to validate results obtained by microarray experiments. For each primer pair, amplification was performed using reagents from the PCR QuantiTect SYBR Green kit (Qiagen) and the Mx3005P thermocycler (Stratagene, La Jolla, CA). After 40 rounds of amplification, the specificity of each primer pair was verified by increasing the temperature from 55°C to 95°C to construct melting curves. Values were expressed by calculating the 2^(-ΔCt) ^value, where the threshold cycle C_t _represents the fractional cycle number at which the fluorescence passes a fixed threshold, and ΔC_t _represents the differences between the C_t _of the gene of interest and that of the housekeeping gene *Rps16*, coding for ribosomal protein S16 sub-unit. Each value represented the average of 3 replicate measurements.

### Genomic sequencing

Genomic DNA was extracted from hearts of adult C57BL/6J and C57.Y^A ^using the *DNeasy blood & tissue *commercial kit (Qiagen Canada, Mississauga, ON). Primers were designed to amplify ~1 kb of upstream promoter, each exon (including 30 bp of intron flanking sequences as well as ~1 kb of the 3'-unstranslated region) of the *Ddx3y *and *Eif2s3y *genes. Amplicons were then sequenced at the McGill Genome Discovery Centre.

## Abbreviations

chrY: chromosome Y; ChrY^C57^: chrY from the C57BL/6J mouse strain; ChrY^A ^: chrY from the A/J mouse strain; CM: cardiomyocyte; CX: castration; FDR: false discovery rate; KEGG: Kyoto Encyclopedia of Genes and Genomes.

## Authors' contributions

BL and RAV contributed equally to the manuscript. All authors were involved in the interpretation of the data and the writing of the manuscript. BL performed all experimental manipulations on animals, the isolation of cardiomyocytes and measurement of their size, extracted RNA from hearts or other tissues, and performed all RT-PCR experiments. RAV and GAC performed the statistical design and analysis of the microarray experiments. CFD was responsible for the overall design, conception and conduct of the study, and coordinated the writing of the manuscript.

## Supplementary Material

Additional file 1 **List of 249 interaction genes**. List of 249 interaction genes (with significant interaction between the effects of strain and surgery). Fold change (FC) was calculated as the ratio of mean expression of either: 1) C57BL/6J over C57.Y^A ^mice (Strain); or 2) Castrated over sham-operated (Treatment) mice. Ratios below 1 are expressed as the negative of the inverse ratio (e.g. a ratio of 0.75 is equivalent to a -1.3 FC). FC equal or higher than 1.5 are formatted **in bold**. For genes represented by multiple probes in the microarray, only the probe with the lowest p-value for the interaction term is included. P-values for the interaction between strain and treatment effects are adjusted for multiple comparisons with an FDR transformation (see methods). Click here for file

Additional file 2 **Differential expression of genes in selected KEGG pathways**. Differential expression of genes in selected KEGG pathways. Fold change (FC) was calculated as the ratio of mean expression in CX vs. sham-operated group. Ratios below 1 are expressed as the negative of the inverse ratio (e.g. a ratio of 0.75 is equivalent to a -1.3 FC). P-values for the interaction between strain and treatment effects are adjusted for multiple comparisons with an FDR transformation (see methods). P-values less than 0.1 are **in bold**. For genes represented by multiple probes in the microarray, only the probe with the lowest p-value for the interaction term is included. Genes are sorted by p-values within pathways. Click here for file

Additional file 3 **Primers used for RT-PCR amplification of chr Y mRNA transcripts**. Primers used for RT-PCR amplification of chr Y mRNA transcripts. Click here for file
