# Supplementary material for: Chromosome Y variants from different inbred mouse strains are linked to differences in the morphologic and molecular responses of cardiac cells to postpubertal testosterone
Source: BMC Genomics. 2009 Apr 7;10:150. doi: 10.1186/1471-2164-10-150 (PMC2679052; doi:10.1186/1471-2164-10-150)
Supplement: Additional file 3 — Primers used for RT-PCR amplification of chr Y mRNA transcripts. Primers used for RT-PCR amplification of chr Y mRNA transcripts. [file 1471-2164-10-150-S3.doc]

| **Gene** | **RefSeq Accession** | **Sense (5’ → 3’)** | **Antisense (5’ → 3’)** | **size (bp)** |
| --- | --- | --- | --- | --- |
| ***Ddx3y*** | BC021453 | gtgccttcttggttggaaag | aatccagcatttgcagaacc | 137 |
| ***Eif2s3y*** | BC043656 | aggaggcagagttagtgcag | cttcttatctggccccaacc | 144 |
| ***Jarid1d*** | AF127244 | tcccaatctagagcgcattc | gaaccaccttttgcctcctc | 116 |
| ***Rbmy1a1*** | NM_011253 | agccaggagaccatcatcac | tgcaaagtgtctcccagaag | 202 |
| ***Sly*** | BC049626 | cgaacgaacgagagaggaag | tccccaagttcatcaaaacc | 234 |
| ***Sry*** | NM_011564 | ttatggtgtggtcccgtggt | ggccttttttcggcttctgt | 124 |
| ***Ssty1*** | X05260 | gtggtctgtgaaaggcaagc | ccagcctcaggattatttgg | 200 |
| ***Ssty2*** | AK006494 | tggtgcctggtctctatcag | cgtttacgccagacacaatg | 205 |
| ***Ube1y1*** | AF150963 | tgtccaagaaacgcaaagtg | acactggaagcctggagatg | 206 |
| ***Usp9y*** | NM_148943 | ggaatggcttggagatgaac | tcttggtcatcagggtcctc | 192 |
| ***Uty*** | AF057367 | atggagaagggcatgagaac | agttggtggtcttggaggtg | 203 |
| ***Zfy1/2*** | X14382 | aggtgttctgggttttcagg | tgcatcagctcctattccatc | 201 |

**Additional file 3:
Primers used for RT-PCR amplification of chr Y mRNA transcripts**

**Supplementary Table S2:
primers used for amplification of *Ddx3y* genomic fragments.**

| **Séquence** | **Sens (5’ → 3’)** | **Antisens (5’ → 3’)** | **Taille (pb)** |
| --- | --- | --- | --- |
| **Prom** | gcaagaaactctttaggggatg | tcagtttaattatccggcttcc | 649 |
| **Prom + ex1** | cctaaatgagtgggctgaaac | tggccaagttaaaacaaagg | 690 |
| **Ex2** | ggtatccagcgtggtttttg | tttcagctcagggtggtttc | 273 |
| **Ex3** | ttaggccttcattcccactg | caaagacttcctgaagggatg | 607 |
| **Ex4** | catttgtagctccctttttgg | cactggatagccattgttgg | 613 |
| **Ex5** | ccatcacaggagaacagtgg | gacccagatgccttaagaagc | 602 |
| **Ex6** | agggtcagaaggatggcttag | tcccctcacaagttgtcctc | 548 |
| **Ex7** | ctcaggtccaatgcaaaagc | ggggaacaaaagagttgctg | 579 |
| **Ex8** | ccttttgagaccttgcttcac | aagccagaaagtaccctggag | 534 |
| **Ex9 & 10** | gggtactttctggcttttgc | tgggaaggataagggtggac | 662 |
| **Ex11 & 12** | cccttatccttcccatttctg | cagacagagacacacacaaaacc | 742 |
| **Ex13 & 14** | agtcagccttgcaaatcctc | tttgaggtcccaagatccag | 578 |
| **Ex15 & 16** | ttctaggccccatggtctac | ccaccaaatcctctgttgtg | 625 |
| **Ex16 & 17** | gcagattcagtggaggatttg | ggtacaaccaagcaggaagtg | 638 |
| **3’UTR1** | ttcatgagagctcaaagtcacaa | aaagcctgctgctgcataat | 791 |
| **3’UTR2** | tatgcagcagcaggctttac | cgtgtgccaccacttcag | 693 |
| **3’UTR3** | tgtgtcaagaacttggttcaga | ttccaattgcctttcattttg | 560 |

Prom : promotor; Ex : exon; 3’UTR : 3’-untranslated region. For fragments where 2 exons are mentioned, the corresponding intron was also amplified.

**Supplementary Table S3:
primers used for amplification of *Eif2s3y* genomic fragments.**

| **Séquence** | **Sens (5’ → 3’)** | **Antisens (5’ → 3’)** | **Taille (pb)** |
| --- | --- | --- | --- |
| **Prom** | ctcgccagtttttggatttg | cagctacttgccatggacttc | 476 |
| **Prom + ex1** | ttgggatggaggttaggatg | cagtcggtatcaccggtctc | 499 |
| **Ex2** | tttctcgaccgctaaaatcc | acacccaagtctcgaggatg | 501 |
| **Ex3 & 4** | agtgtctcaggctcacagagg | ggctactaagcaactgtcacatc | 728 |
| **Ex5** | gatcatcccctttccacctc | aaccaagttcaatgcttctgg | 486 |
| **Ex6** | tcttgggtcttggtttcacc | caccataaattctgtgtttggatg | 457 |
| **Ex7** | gtcagtgcactcaaccctca | gtggtgcatgcctttaatcc | 502 |
| **Ex8** | gctggcctcaaactctgaaa | ccagagagatccaacttctggta | 510 |
| **Ex9** | gcttgtgcaccaataccatc | tggacaaatagccctttgaatac | 531 |
| **Ex10** | tctacaggggaccaaatgag | agagcaccgactgttcttcc | 524 |
| **Ex11** | cgtggatccatacaccagac | ccagattgcaaagtgaagacc | 587 |
| **Ex12** | ccttcttctggcctctaccg | tgaggcaaaattgaatccag | 663 |

Prom : promotor; Ex : exon; 3’UTR : 3’-untranslated region. For fragments where 2 exons are mentioned, the corresponding intron was also amplified.
